# Ni endoscopic classification for Storz Professional Image Enhancement System (SPIES) endoscopy in the detection of upper aerodigestive tract (UADT) tumours

**DOI:** 10.1038/s41598-020-64011-6

**Published:** 2020-04-24

**Authors:** Baharudin Abdullah, Nurul Syeha Abdull Rasid, Norhafiza Mat Lazim, Veronika Volgger, Christian Stephan Betz, Zahiruddin Wan Mohammad, Nik Fariza Husna Nik Hassan

**Affiliations:** 1 Department of Otorhinolaryngology Head and Neck Surgery, School of Medical Sciences, Universiti Sains Malaysia, 16150 Kubang Kerian Kelantan, Malaysia; 2 Department of Otorhinolaryngology Head & Neck Surgery, Klinikum der Universität München, 81377 Munich, Germany; 3 Department of Otorhinolaryngology, Universität sklinikum Hamburg-Eppendorf Head- and Neuro-Centre, Martinistraße 52, Building O10, 20246 Hamburg, Germany; 4 Department of Community Medicine, School of Medical Sciences, Universiti Sains Malaysia, 16150 Kubang Kerian Kelantan, Malaysia

**Keywords:** Diseases, Medical research

## Abstract

The diagnostic procedure for upper aerodigestive tract (UADT) tumours is by white light endoscopy (WLE) combined with biopsy. However, WLE has difficulty identifying minute epithelial changes which hinders early diagnosis. Storz Professional Image Enhancement System (SPIES) is designed to enhance the visualization of microvasculature on the mucosal surface and detect any epithelial changes. In this study, we aimed to evaluate the use of Ni endoscopic classification with SPIES endoscopy in the detection of UADT tumours. Fifty-nine patients with suspected UADT tumours underwent WLE followed by SPIES endoscopy. All the tumours were biopsied and sent for histopathological examination (HPE). The kappa index (κ) was used to evaluate the agreement between the methods. The level of agreement between SPIES using Ni classification and HPE showed almost perfect agreement as compared to moderate agreement between WLE and HPE. The sensitivity and specificity for WLE and HPE were 77.5% and 84.2% respectively with positive predictive value (PPV) of 91.2% and negative predictive value (NPV) of 64%. The sensitivity and specificity for SPIES endoscopy using Ni classification and HPE were 97.5% and 94.7% respectively with PPV of 97.5% and NPV of 94.7%. SPIES endoscopy using Ni classification is a valid tool for earlier tumour detection.

## Introduction

The gold standard in diagnosing upper aerodigestive tract (UADT) tumours is by identification of suspicious lesions by white light endoscopy (WLE) combined with the histopathological examination (HPE) findings. However, WLE can only provide poor quality images causing difficulty to identify minute epithelial changes and making it difficult to differentiate benign from malignant tumours. Neoangiogenesis is a prerequisite for the progression of precancerous and cancerous lesions of the UADT^1^. Subepithelial and epithelial microvascular irregularities will appear due to alterations of the vessel architecture and it depends on the degree of dysplasia. The superficial changes of neoangiogenesis created by the UADT lesions can be enhanced and recognized by using narrow band imaging (NBI) and Storz Professional Image Enhancement System (SPIES)^1,2^.

SPIES (Karl Storz, Tuttlingen, Germany) uses a high definition camera system with image enhanced endoscopy for better endoscopic visualization. In the assessment of the microvasculature’s pattern on the mucosal surface, the endoscopic images can be visualized by using 5 spectral modes of “Clara, Chroma, combined Clara and Chroma, Spectra A and Spectra B”^1^. While Clara and Chroma modes are used mainly for endoscopic procedures to gain better clarity of anatomical structures, Spectra A and Spectra B modes are used to visualize the vascular arrangements in the mucosa of UADT. SPIES endoscopy aids in visualizing early changes of the vascular arrangements and improves the diagnostic capability in early cancer detection. Early detection of malignant change improves the prognosis as immediate definitive treatment can be instituted. In comparison to NBI (Olympus Corporation, Tokyo, Japan), SPIES uses the different light wavelengths obtained with the standard white light sources to enhance the image quality^1,2^. The technological difference between NBI and SPIES purely relies on the different design of the light filtering process. Although both use filter wavelengths, pre-processing of image occurs in NBI, while in SPIES it happens in the post-processing phase. Additionally, NBI uses one kind of filter, while SPIES uses a software system to create different light wavelengths and contrast in the production of images. SPIES endoscopy is a useful technique to obtain clearer images and reduces the chances of missed diagnosis. It also enables complete resection of tumour by delineating the margin of healthy and tumour tissue.

The presence of dilated and abnormal intraepithelial capillary loops in a brownish area with dark spots is the typical finding of NBI in patients with precancerous lesions^3^. There are two main classifications based on the epithelial microvascular changes being used for NBI endoscopy to differentiate benign and malignant lesions of UADT. By identifying the capillary loop arrangements on the mucosal vessels of the oral cavity, Takano *et al*.^4^ classified their findings as 4 types; type 1 when there is presence of “normal mucosa and regular brown dots”, type 2 when there is “capillary dilation and crossing”, type 3 when there is “capillary elongation and meandering” and type 4 when there is “capillary destruction and angiogenesis”. Their classification is actually a modification of Inoue classification^5^, which is a classification based on microvascular changes seen on the pharyngoesophageal epithelium. However, the most used classification system of vascular changes seen in laryngeal and pharyngeal lesions is the Ni classification^6,7^ based on the microvasculature pattern of the abnormal capillary loop arrangements. Ni classification^6^ categorized the microvascular changes into five different types; types I to III demonstrating longitudinal oblique or branching vessels but no intraepithelial papillary capillary loop, while types IV and V representing visible perpendicular intraepithelial papillary capillary loops with increasing irregularity. Type I-IV are considered as benign lesion while type V is considered as malignant lesion^6,7^. Bertino *et al*.^8^ compared both classifications for NBI endoscopy and found that Ni^6^ has better sensitivity and specificity as compared to modified Inoue classification^4^ in detecting benign and malignant lesions of UADT.

It has been established that both NBI and SPIES endoscopy systems are comparable in recognition and analysis of vascular patterns that is typical for benign and malignant lesions^9^. Thus, any classifications used and valid for NBI should also be applicable for SPIES as well. However, there was no study done on the concordance and validation of the Ni classification system for SPIES endoscopy application. There was also no previous study done using SPIES endoscopy with Ni classification system to identify UADT tumours as most studies were specifically for laryngeal and hypopharyngeal lesions. Therefore, we aimed to use and validate the Ni endoscopic classification with SPIES endoscopy system in the detection of UADT tumours.

## Methods

### Study population and procedures

**Figure 1:**
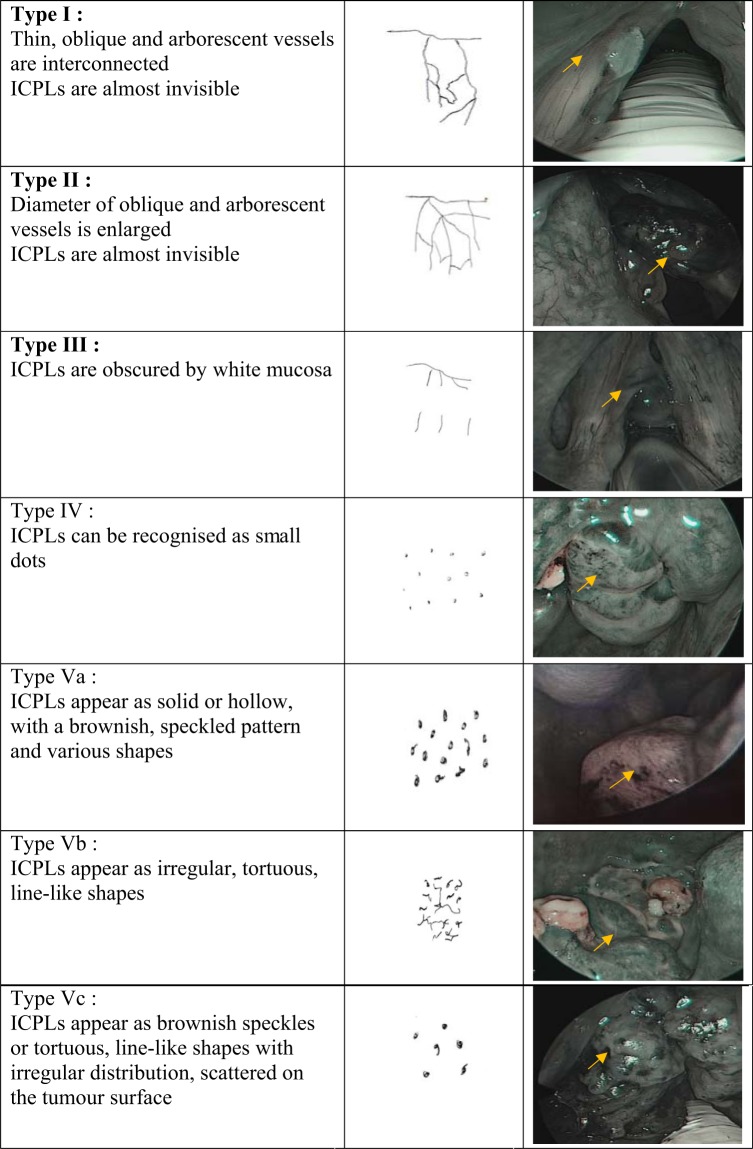
Ni classification: type I-IV (benign lesion); type Va-Vc (malignant lesion).

The study protocol was approved by Human Research Ethics Committee USM [USM/JEPeM/16110492], performed in adherence with the Declaration of Helsinki and approved guidelines. Patients who attended the otorhinolaryngology clinic were recruited from March 2017 until March 2018. Inclusion criteria were patients aged 18 years old and above with suspicion of UADT tumours including sinonasal, nasopharygeal, oral, oropharyngeal or laryngeal tumours. Patients who had undergone surgery involving the UADT, prior treatment with chemoradiotherapy and pregnant women were excluded. Informed consent was obtained from all patients before their participation in this study. The patients’ demographic data and clinical history were recorded in the patient’s proforma. All patients with suspected UADT tumours underwent both WLE and SPIES endoscopic examination. WLE was performed initially, followed by SPIES endoscopy, which was easily switched between each modality by using a button on the camera head. SPIES endoscopy in a Spectra A mode was performed using 0 degree or 70 degrees, 4 mm rigid endoscope (Karl Storz, Tuttlingen, Germany) and connected to Image 1 high-definition camera system (Karl Storz, Tuttlingen, Germany). The UADT was locally anaesthetized before introducing the rigid endoscope. 10% cocaine hydrochloride solution was used as a topical anaesthetic, with vasoconstrictors for nasal endoscopic procedures while 10% lignocaine spray was used for oral or laryngeal endoscopic examination. The procedure was done under local anaesthesia except for patients who could not tolerate the procedure or patients with laryngeal mass who required biopsy under general anaesthesia. The videos of all endoscopic examinations were recorded and saved on a personal computer. On WLE, lesions were considered suspicious for cancer when there were changes such as leucoplakia, erythroplakia, exophytic, or ulcerated lesions seen. Then, the SPIES endoscopy findings were evaluated. The first evaluation was made according to modified Inohue classification^4^ and the second according to the classification of the microvascular endoscopic patterns of Ni classification^6^ (Fig. 1). The modified Inohue classification^4^ classifies type I-II as benign and type III-IV as malignant while for Ni classification^6^, type I-IV indicates a benign lesion and type Va-Vc indicates a malignant lesion. Both WLE and SPIES endoscopy were performed by three authors (BA, NSAR, NML). Biopsy of the mass was taken, fixed in 4% formalin, and submitted for HPE. The HPE results were compared with WLE and SPIES endoscopic findings.

### Outcome measures and statistics

Data were stored in a computer based filling system, and analysed using the PASW Statistics 22 software (SPSS, IBM, USA). The kappa index (κ) was used to evaluate the agreement of WLE or SPIES results with HPE findings and agreement between the methods. The value of kappa less than 0.20 is considered as no agreement, 0.21 to 0.39 as minimal, 0.40 to 0.59 as weak, 0.60 to 0.79 as moderate, 0.80 to 0.90 as strong and above 0.90 as almost perfect agreement. The sensitivity, specificity, and positive and negative predictive values for WLE and SPIES endoscopy were calculated. A p value of < 0.05 was considered statistically significant.

## Results

A total of 59 patients,41 males and 18 females, were enrolled with the mean age of 52.2 years. The youngest was 18 years old and the oldest was 94 years old. There were 19 lesions from the larynx, 17 lesions from the nasal cavity, 11 lesions from the nasopharynx, 9 lesions from the oral cavity and 3 lesions from the oropharynx. The demography is shown in Table 1.

**Table 1:** Characteristics of patients recruited for the study.

Variables	n (%)
**Gender**	
Male	41 (69.5)
Female	18 (30.5)
**Age (year)**	
Mean (SD)	52.2 (16.5)
Range (min – max)	18–94
**Race**	
Malay	44 (74.6)
Chinese	11 (18.6)
Indian	4 (6.8)
**Smoking status**	
Yes	19 (32.2)
No	40 (67.8)
**Site of lesions**	
Nasal cavity	17 (28.8)
Nasopharynx	11 (18.6)
Oral cavity	9 (15.3)
Oropharynx	3 (5.1)
Larynx	19 (32.2)
**Histopathological examination (HPE)**	
Benign	40 (69.8)
Malignant	19 (32.2)

### Comparison between histology and WLE or SPIES optical findings

Based on the HPE findings, there were 40 benign lesions and 19 malignant lesions from a total of 59 lesions (Table 2). Out of 40 benign lesions, 12 patients had lymphoid hyperplasia and 9 patients had inflammatory nasal polyps. For benign lesions, SPIES endoscopy revealed only one false positive result which was a granulation tissue. As for malignancy, 18 lesions were laryngeal carcinoma (8 patients), oral cavity carcinoma (6 patients), nasal cavity carcinoma (3 patients) and nasopharyngeal carcinoma (1 patient). All lesions with carcinoma correlate with SPIES findings except for one false negative result which was a case of a diffuse B cell lymphoma (1 patient). SPIES endoscopy was able to detect 39 benign lesions correctly out of a total of 40 lesions (97.5%) and 18 malignant lesions from a total of 19 lesions (94.7%). In contrast, WLE was able to identify only 31 lesions out of 40 benign lesions and 16 lesions out of 19 malignant lesions.

**Table 2:** Microvasculature characteristics of tumours by Ni classification.

Histology	Ni classification (Numbers of patients)						
	Benign				Malignant		
	I	II	III	IV	Va	Vb	Vc
**Benign findings**							
Squamous ulceration of tonsil				1			
Granulation tissue		1	1	1	1		
Tonsillar lymphangiomatous polyp	1						
Benign laryngeal nodule		1	1				
Fibrolipoma		1					
Capillary hemangioma				2			
Lymphoid hyperplasia	1	2	7	2			
Keratosis of vocal cord	1						
Inflammatory nasal polyp	2	2	3	2			
Laryngeal polyp	1	1					
Vocal cord papilloma			1	1			
Angiofibroma				1			
Inverted papilloma			1				
Severe dysplasia of vocal cord		1					
**Malignant findings**							
SCC of larynx					2	3	3
SCC of nasal cavity					1		2
SCC of nasopharynx							1
SCC of oral cavity					1	2	3
Diffuse B cell lymphoma			1				

^*^SCC = Squamous cell carcinoma.

### Statistical analysis

Results of WLE and SPIES endoscopy (modified Inohue and Ni classifications) with the HPE were compared. The level of agreement between WLE and histological assessment was 78.3%, kappa index *κ* = 0.592 (95% CI 0.368–0.755)(*p* < 0.001) which showed moderate agreement. The level of agreement between SPIES endoscopy and histological assessment was 86.7%, kappa index *κ* = 0.753 (95% CI 0.482–0.916) (*p* < 0.001) for modified Inohue classification and the agreement was 95.0%, kappa index *κ* = 0.927 (95% CI 0.762–1.000) (*p* < 0.001) for Ni classification. The agreement was confirmed as strong for modified Inohue classification while almost perfect agreement for Ni classification.

The sensitivity and specificity in the differentiation between WLE and HPE were 77.5% and 84.2% respectively with positive predictive value of 91.2% and negative predictive value of 64%. The sensitivity in the differentiation between SPIES endoscopy using modified Inohue classification and HPE was 87.5% while specificity was 89.5% with positive predictive value of 94.6% and negative predictive value of 77.3% (Table 3). The differentiation between SPIES endoscopy using Ni classification and HPE demonstrated a sensitivity of 97.5% and specificity of 94.7% with positive predictive value of 97.5% and negative predictive value of 94.7% (Table 3).

**Table 3:** Diagnostic performance of white light endoscopy and SPIES using Modified Inohue classification and Ni classification for the detection of tumours in comparison with histopathological findings.

	White light endoscopy		Modified Inohue		Ni classification	
		(95% CI)		(95% CI)		(95% CI)
Sensitivity	84.2	(74.9–93.5)	87.5	(79.1–95.9)	94.7	(89.0–100)
Specificity	77.5	(66.8–88.7)	89.5	(81.6–97.3)	97.5	(93.5–100)
PPV	64.0	(51.7–76.3)	94.6	(88.8–100)	94.7	(89.0–100)
NPV	91.2	(83.9–98.4)	77.3	(66.6–88.0)	97.5	(93.5–100)
Kappa index, *κ*	0.6	(0.4–0.8)	0.8	(0.5–0.9)	0.9	(0.8–1.0)

κ value:<0.20 (no agreement), 0.21 to 0.39 (minimal), 0.40 to 0.59 (weak), 0.60 to 0.79 (moderate), 0.80 to 0.90 (strong), above 0.90 (almost perfect agreement).

## Discussion

Patients with UADT tumour are routinely examined by WLE which is the commonest method to obtain a detailed evaluation of the superficial extent of a squamous cell cancer arising within the UADT. In the early stages, UADT tumours are indistinguishable from normal tissue and very difficult to detect by conventional WLE. Hence, early recognition of tumour by the treating physician may not be possible. In addition, biopsy in early carcinoma is difficult, because there is minimal mucosal change making it hard to distinguish between normal and tumour tissue for a proper specimen to be taken. Technological improvements in endoscopy is the key to improve the early diagnosis of UADT tumours.

The features and organisation of blood vessels are dynamic and may undergo change during the transition from a precancerous to a malignant state, as the microvascular architecture becomes more dilated, elongated, distorted, or replaced by neoplastic vasculature due to the epithelial cancerogenetic stimulus^1,6,10^. The alterations of the vessel architecture become more obvious in areas of severe dysplasia when compared to mild dysplasia^1^. The Committee on Endoscopic Laryngeal Imaging of the European Laryngological Society (ELS) has proposed an approach to distinguish between benign (longitudinal vessels) and malignant (perpendicular vessels) lesions instead of a classification of vascular pattern correlated with their histopathologic findings done by Ni and colleagues^6,7,10,11^. Nevertheless, the Ni classification and the ELS classification are almost similar, as the Ni types I, II, and III represent changes in the longitudinal vessels, whereas the Ni types IV and V represent the perpendicular vessels for ELS classification^7^. Among the classifications, ELS is the easiest and more practical to be taught, remembered, and systematically applied.

Our data showed SPIES endoscopy using Ni classification^6^ demonstrate a high sensitivity of 97.5% and specificity of 94.7%, with positive and negative predictive values of 97.5%, and 94.7% respectively in the detection of UADT tumours. In comparison, the conventional WLE has a sensitivity of 77.5% and a specificity of 84.2% whereas SPIES endoscopy using modified Inohue classification has a sensitivity of 87.5% and a specificity of 89.5%. Our findings on Ni classification^6^ used with SPIES endoscopy are consistent with another study that assessed its use with NBI endoscopy^8^. As there was an almost perfect agreement of SPIES endoscopy using Ni classification^6^ with the HPE results (*κ* = 0.927, *p* < 0.001), SPIES endoscopy is comparable to the results obtained by NBI endoscopy in the early assessment of UADT tumours. Hence, our results showed that both NBI and SPIES have equal property in picking up and diagnosing UADT tumours.

In our study, SPIES endoscopy revealed only one false positive result in the case of granulation tissue and one false negative result in the case of diffuse B cell lymphoma. In the case of granulation tissue, it becomes falsely interpreted as malignancy due to the presence of necrotic tissue that obscured the lesion. In the case of diffuse B cell lymphoma, there was no change of the microvascular pattern within the epithelial and mucosal lining and this was rightly diagnosed as normal epithelium. Ni *et al*. reported that in the assessment of intraepithelial papillary capillary loop features using NBI endoscopy, interference is possible in the presence of necrotic tissue or a thick white patch overlying the lesions, resulting in false negative findings^3,6^. Therefore, surrounding regions and areas with a thinner white patch need to be evaluated, in order to delineate any dots or irregular, tortuous, line-like shapes which may aid classification^6^. In addition, bleeding during the rigid endoscope insertion may affect the evaluation of intraepithelial papillary capillary loop features. The presence of blood on mucosa will be shown as generalized black lesion as haemoglobin absorbs blue light. Thus, touching and injuring the mucosal surface should be avoided during the endoscopic procedure. The result of our study showed, that SPIES endoscopy in combination with Ni classification^6^ could detect accurately all squamous cell carcinomas but not diffuse B cell lymphoma as there was no alteration of the microvascular pattern within the epithelial and mucosal lining of the lesion.

One of the recommendations by National Institute for Health and Care Excellence (NICE)^12^ is to emphasize the role of NBI endoscopy as their strategy for an earlier tumour detection. As both NBI endoscopy and SPIES endoscopy methods are comparable in recognition and analysis of benign and malignant lesions^9^, SPIES endoscopy should also be recommended as one of the strategy for an earlier diagnosis of UADT tumours. Both NBI and SPIES endoscopy systems form an optimal strategy for preventing the progression of the cancer and improving the prognosis by earlier detection. Furthermore, early detection may help in minimally invasive treatment such as endoscopic resection with curative intent. Watanabe *et al*.^13^ conducted a study on patients with the suspicion of having laryngeal cancer and they found that NBI was superior in the early detection of abnormal microvascular changes when compared to WLE. Their study reported a sensitivity of 91% and specificity of 92% in the differentiation between low-grade and high-grade dysplasia^13^. Another study by Piazza *et al*.^14^ on patients undergoing treatment or follow-up for laryngeal cancer, revealed overall sensitivity, specificity, positive predictive value and negative predictive value of 98%, 90%, 86%, and 88% respectively. The outcome of their studies using NBI endoscopy are comparable to our results with SPIES endoscopy.

## Conclusions

Ni classification is valid to be used for SPIES endoscopy in the distinction between benign and malignant tumours. It offers a high sensitivity and specificity rate in the detection of tumours and a reliable diagnostic tool for earlier UADT tumours recognition.

## Data Availability

The datasets generated during and/or analysed during the current study are available from the corresponding author on reasonable request.
